# Clinicopathological Characterization of Pediatric Atypical Teratoid/Rhabdoid Tumors and an HE–IHC Dual-Path Deep Learning Model for Auxiliary Diagnosis

**DOI:** 10.3390/diagnostics16101515

**Published:** 2026-05-16

**Authors:** Jian Tian, Nan Zhang, Zhijuan Deng, Jianwen Wang, Wentao Zheng

**Affiliations:** 1 School of Economics and Management, University of Science and Technology Beijing, Beijing 100083, China; tianjian72@hotmail.com; 2 Department of Pathology, Beijing Children’s Hospital, Capital Medical University, National Center for Children’s Health, Beijing 100045, China; dengzhijuan0825@sina.com (Z.D.); dopwang@sina.com (J.W.); zhengwentao1029@sina.com (W.Z.)

**Keywords:** atypical teratoid/rhabdoid tumor, children, clinicopathology, FISH, dual-path AI model, auxiliary diagnosis, transfer learning

## Abstract

**Background/Objectives:** Atypical teratoid/rhabdoid tumor (AT/RT) is a rare and aggressive pediatric embryonal tumor of the central nervous system with marked histological and immunophenotypic heterogeneity, which can make diagnosis difficult in some cases. This study aimed to summarize the clinicopathological and molecular features of pediatric AT/RT and to evaluate an HE–IHC dual-path deep learning model as an auxiliary diagnostic approach. **Methods:** Clinical, histopathological, immunophenotypic, ultrastructural, and fluorescence in situ hybridization (FISH) data were retrospectively collected from 18 children with AT/RT treated at Beijing Children’s Hospital between February 2010 and April 2021. A total of 361 pathological images were used to train and test a ResNet50-based dual-path classification model with transfer learning and feature fusion. An additional independent test set of 175 histological and immunohistochemical images from six newly collected patients was used for supplementary validation. **Results:** The mean age at diagnosis was 2 years and 3 months. All cases showed loss of INI1 expression, positivity for CK and EMA, and a high Ki-67 index. FISH analysis identified SMARCB1 deletion in 7 of 15 tested cases. In the original image-based test set, the dual-path model achieved an accuracy of 90.91%, compared with 81.82% for the model without transfer learning, 86.36% for the single-path immunohistochemistry model, and 50.00% for the single-path histological model. In the additional independent test set, the trained model correctly classified all 175 images. **Conclusions:** Pediatric AT/RT shows diverse clinicopathological features and complex SMARCB1 alteration patterns. The HE–IHC dual-path model showed encouraging preliminary performance for auxiliary pathological assessment; however, larger multicenter cohorts with molecular subgroup annotation are needed for further validation before routine clinical application.

## 1. Introduction

Atypical teratoid/rhabdoid tumor (AT/RT) is a rare and highly malignant embryonal tumor of the central nervous system. Recent clinical and population-based studies have continued to show that it occurs predominantly in very young children, has marked clinicopathological heterogeneity, and remains associated with an unfavorable prognosis in many patients [1,2,3]. Histologically, AT/RT displays diverse morphologic patterns, and diagnostically difficult cases may overlap with other embryonal neoplasms, which remains a practical challenge in surgical pathology [4,5]. Recent work has also emphasized that molecular grouping is closely related to pathological features, prognosis, and future therapeutic stratification [6,7]. Earlier reviews likewise summarized the biological basis, diagnostic challenges, SMARCB1-related mechanisms, and emerging targeted therapeutic directions for pediatric AT/RT [8,9,10,11].

SMARCB1 alteration remains the central molecular event in most AT/RTs, whereas a small subset shows SMARCA4 deficiency. In routine practice, laboratory diagnosis still relies on combined assessment of morphology, immunohistochemistry, and molecular testing [4,5]. In the present study, fluorescence in situ hybridization was used to evaluate SMARCB1 (22q11) deletion in 15 cases in order to further characterize the molecular pathological features of AT/RT. Given the histological diversity and potential diagnostic difficulty of AT/RT, traditional diagnostic methods rely heavily on pathologists’ professional experience, which may lead to misdiagnosis, especially in medical institutions with limited pediatric pathology expertise. Histological images can reflect morphological features such as rhabdoid cells and small-blue-round cells, while immunohistochemistry (IHC) images can provide specific marker information, such as INI1 loss and CK/EMA positivity, which are complementary for AT/RT diagnosis. To address this need, we developed the HE–IHC Dual-Path Deep Learning Model, also referred to as the HE–IHC Dual-Path Model, which integrates these two types of image information. The model adopts ResNet50 as the feature extraction backbone and applies transfer learning and data augmentation techniques to overcome the limitation of small medical image sample sizes, aiming to provide an objective, efficient, and high-accuracy auxiliary diagnostic tool for AT/RT [12,13].

## 2. Materials and Methods

### 2.1. Ethical Approval

This study was approved by the Ethics Committee of Beijing Children’s Hospital (approval number: [2021]-E-077-R; approval date: 14 May 2021). This was a retrospective analysis, and the requirement for informed consent was waived.

### 2.2. Materials

Eighteen AT/RT cases admitted to Beijing Children’s Hospital from February 2010 to April 2021 were retrospectively analyzed. All patients underwent surgery, and the diagnoses were independently reviewed and confirmed by two senior pediatric pathologists (N.Z. and Z.D.). All diagnoses were based on WHO Classification of Tumours of the Central Nervous System standards.

For the HE–IHC Dual-Path Model, a total of 361 image files were collected from the hospital’s pathological archive, including 233 AT/RT cases and 128 non-AT/RT cases (serving as controls). The AT/RT group included 69 IHC images (immunohistochemical staining targeting INI1, CK, EMA, etc.) and 164 histological images (HE-stained sections). The non-AT/RT control group consisted of 30 IHC images (with positive INI1 expression, consistent with non-AT/RT immunophenotypic characteristics) and 98 histological images (including medulloblastoma, high-grade glioma, and other tumors that need to be differentiated from AT/RT). All images were anonymized to comply with ethical requirements and data security regulations.

### 2.3. Methods

#### 2.3.1. Diagnostic Criteria for AT/RT

All tumors were diagnosed as AT/RT according to the WHO Classification of Tumors of the Central Nervous System, 5th edition, based on integrated histopathological and immunophenotypic assessment. All cases were reviewed as central nervous system embryonal tumors with polyphenotypic features, and all showed complete loss of nuclear INI1/SMARCB1 expression by immunohistochemistry, which served as the key diagnostic surrogate marker. SMARCB1 fluorescence in situ hybridization (FISH) was performed only as an adjunct molecular assay in the subset of cases with sufficient formalin-fixed paraffin-embedded tissue and was not used as the sole diagnostic criterion [5,14].

#### 2.3.2. Hematoxylin/Eosin (HE) Staining

Eighteen tumor specimens were fixed in 10% neutral buffered formalin, routinely dehydrated, embedded in paraffin, sectioned at 4 µm, and stained with hematoxylin and eosin.

#### 2.3.3. Immunohistochemical Staining

Immunohistochemical staining was performed using the SP method and DAB color reaction. Antibodies used include smooth muscle actin (SMA), S-100 protein, Desmin, synaptophysin (Syn), NeuN, cytokeratin (CK (Pan), AE1/AE3), epithelial membrane antigen (EMA), CD99, INI-1, glial fibrillary acidic protein (GFAP), Olig2, H3K27M, Olig2 and Ki-67. The antibodies were purchased from Beijing Zhongshan Jinqiao Biotechnology Co., Ltd. (Beijing, China), and the dyeing procedures were carried out according to the product instructions.

#### 2.3.4. Fluorescence In Situ Hybridization

SMARCB1 (22q11) gene was detected in 15 samples by fluorescence in situ hybridization (FISH). The SMARCB1 (22q11) gene deletion probe was purchased from Guangzhou Ambiping Pharmaceutical Technology Co., Ltd. (Guangzhou, China). The SMARCB1 (22q11) gene deletion probe was fluorescently labeled at the SMARCB1 locus in red and at the 22q12 locus in green.

#### 2.3.5. Electron Microscope

In 2 cases, 3–4 pieces of fresh tumor specimens were taken, fixed with 3% glutaraldehyde and 1% osmium acid, and embedded with epoxy resin 812. Semi-thin sections were located, ultra-thin sections were stained with uranium and lead, and the specimens were observed using a JEM-1400 transmission electron microscope (JEOL Ltd., Tokyo, Japan).

#### 2.3.6. HE–IHC Dual-Path Model Development and Training

**Figure 1:**
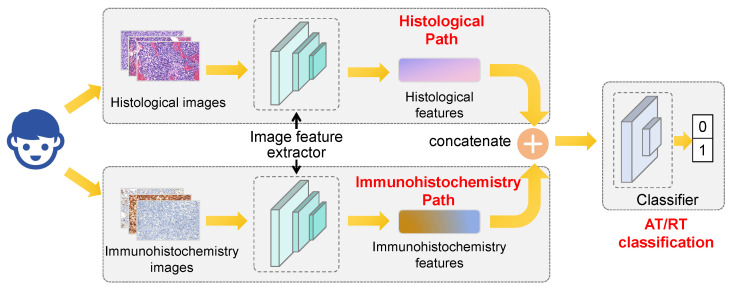
Hematoxylin and eosin–immunohistochemistry (HE–IHC) Dual-Path Model architecture. Two ResNet50 branches extract histological and immunohistochemical features, which are concatenated for atypical teratoid/rhabdoid tumor (AT/RT) classification.

Model Architecture: The overall HE–IHC Dual-Path Model architecture is illustrated in Figure 1. Briefly, the HE–IHC Dual-Path Model contains two parallel feature-extraction branches that take histological images and immunohistochemistry (IHC) images as inputs, respectively. Each branch uses a ResNet50 backbone with transfer learning to extract high-level features. The features from the two branches are then fused by concatenation and fed into a lightweight classifier with fully connected layers to output the probability of AT/RT versus non-AT/RT. Dropout is applied in the classifier to reduce overfitting.

Data Preprocessing and Augmentation: All images were resized to a uniform size of 224×224 pixels. For the training set, data augmentation techniques were applied to expand the sample diversity: random resized cropping with a scale range of 0.8–1.0, random horizontal flipping, random rotation of $\pm 15^{\circ }$, and color jitter for brightness/contrast adjustment with a range of 0.2. Both training and test sets were normalized using the mean [0.485, 0.456, 0.406] and standard deviation [0.229, 0.224, 0.225] of the ImageNet dataset to align with the pre-training data distribution of ResNet50.

Transfer Learning Strategy: Considering the limited number of medical image samples, transfer learning was adopted. Transfer learning is well-suited to small-sample settings because knowledge learned from a large source domain can be transferred to a target task with limited labeled data [15]. The ResNet50 backbones were pre-trained on the large-scale ImageNet dataset to obtain general image feature extraction capabilities. During fine-tuning on the AT/RT image dataset, the parameters of the ResNet50 backbones were frozen first, and only the parameters of the feature fusion module were trained. After 5 epochs of training, the backbone parameters were unfrozen with a reduced learning rate (1 × 10^−5^) for joint fine-tuning to adapt to medical image features.

Training Configuration: The model was implemented based on the PyTorch framework, version 2.0.1, and trained on a workstation equipped with an Intel Core i5-14600KF CPU, an NVIDIA GeForce RTX 4070 Ti SUPER GPU, 64 GB DDR5 memory, and a 1 TB NVMe SSD. The batch size was set to 8, the optimizer was Adam with an initial learning rate of 0.001 and weight decay of 1 × 10^−5^, and the loss function was cross-entropy loss. The dataset was split into training, 80%, and test, 20%, sets using stratified sampling. The total number of training epochs was 10, and the model with the highest test accuracy was saved as the best model.

Performance Evaluation: The primary evaluation metric was classification accuracy. Control experiments included: (1) dual-path model without transfer learning; (2) single-path model using only immunohistochemistry images; (3) single-path model using only histological images. All control models adopted the same training configuration except for structural or strategy differences.

Additional Independent Validation: To further assess generalizability, we assembled an additional independent test set consisting of 175 newly collected histological and immunohistochemical images from six patients who were not included in model training, model selection, or the original test set. The best-performing trained model was directly applied to this additional cohort without retraining or parameter adjustment.

## 3. Results

### 3.1. Clinical Characteristics and Imaging Findings

**Figure 2:**
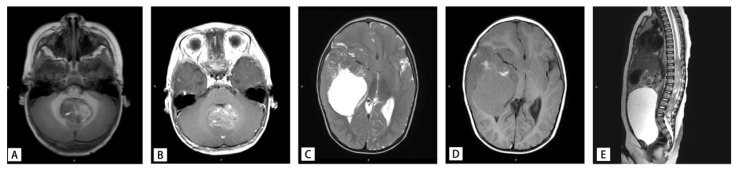
Atypical teratoid/rhabdoid tumor (AT/RT) magnetic resonance imaging. (**A**,**B**) Infratentorial tumor on T1-weighted imaging (T1WI) and enhanced T1-weighted imaging (T1WI). (**C**,**D**) Supratentorial tumor on T2-weighted imaging (T2WI) and T1-weighted imaging (T1WI). (**E**) Spinal cord tumor on T2-weighted imaging (T2WI).

Of the 18 patients, 8 were boys and 10 were girls. The age of diagnosis ranged from 5 months to 6 years and 7 months. The mean age of diagnosis was 2 years and 3 months. Five tumors were situated in supratentorial (3 in temporal lobe, 1 in suprasellar, 1 in basal ganglia), 10 in infratentorial, and 3 in spinal cord. The clinical symptoms were related to the sites of the tumors. The clinical and pathological characteristics of the 18 pediatric AT/RT patients are summarized in Table 1. MR imaging showed significant mass effect with heterogeneous enhancement in all patients (Figure 2). According to the MR imaging results, it was easy to misdiagnose infratentorial tumors as medulloblastoma, while supratentorial and spinal tumors were easily misidentified as high-grade glioma or anaplastic ependyma.

### 3.2. Pathological Examination

**Figure 3:**
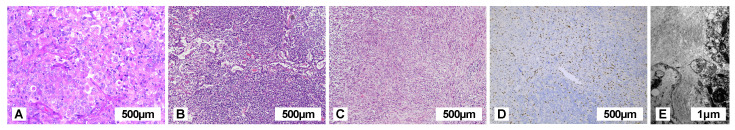
Histological and immunohistochemical features of atypical teratoid/rhabdoid tumor (AT/RT). (**A**) Classical rhabdoid cells. (**B**) Small-blue-round tumor cells. (**C**) Spindle-shaped tumor cells. (**D**) Streptavidin–peroxidase (SP) immunostaining showing loss of integrase interactor 1 (INI1) expression. (**E**) Transmission electron microscopy showing intermediate filaments.

Grossly, the tumor had a solid and grayish-pink appearance, with grayish-yellow necrosis and brown bleeding areas in local areas. Microscopically, 12 cases of the tumor showed rhabdoid tumor cells (Figure 3A), and the tumor cells were medium-large sized, round, or ovoid. These cells had typical vesicular nuclei with obvious nucleoli, abundant and eosinophilic cytoplasm, some even with visible intracytoplasmic homogenization inclusion body. In these 12 cases, spindle mesenchymal cell differentiation was observed in 1 case, with primitive neuroectodermal small-blue-round tumor cells in 5 cases, and 1 case with both. The remaining six tumors presented as small-blue-round primitive neuroectodermal tumor cells without rhabdoid cells; one tumor showed adenoid and ribbon-like structures with epithelial differentiation (Figure 3B), and two tumors showed spindle mesenchymal cell differentiation (Figure 3C).

### 3.3. Immunohistochemical Staining Results

In all cases, the tumor cells exhibited positive CK and EMA expression, which was located at the cell membrane of the focal tumor. The positive rates for SYN, S-100, SMA, Desmin, and CD99 were 52.9%, 52.9%, 47.1%, 29.4%, and 17.6%, respectively, whereas the positive rates for GFAP and CD34 were 5.7%. NeuN, Olig2, and H3K27M were all expressed negatively. The proliferation index for Ki-67 was high, ranging from 40 to 90%. In all cases, the tumor cells lost nuclear expression of INI1 (Figure 3D).

### 3.4. Electron Microscope Results

The transmission electron microscope was performed in 2 tumor specimens, both of which showed the inclusion of bodies of intermediate filaments in the cytoplasm of tumor cells (Figure 3E). They were arranged in parallel or intertwined into clumps, without microtubules and neurites. A large number of intermediate filaments were also observed between cells, and no junction complex was observed between tumor cells.

### 3.5. Fluorescence in Situ Hybridization (FISH) Results

**Figure 4:**
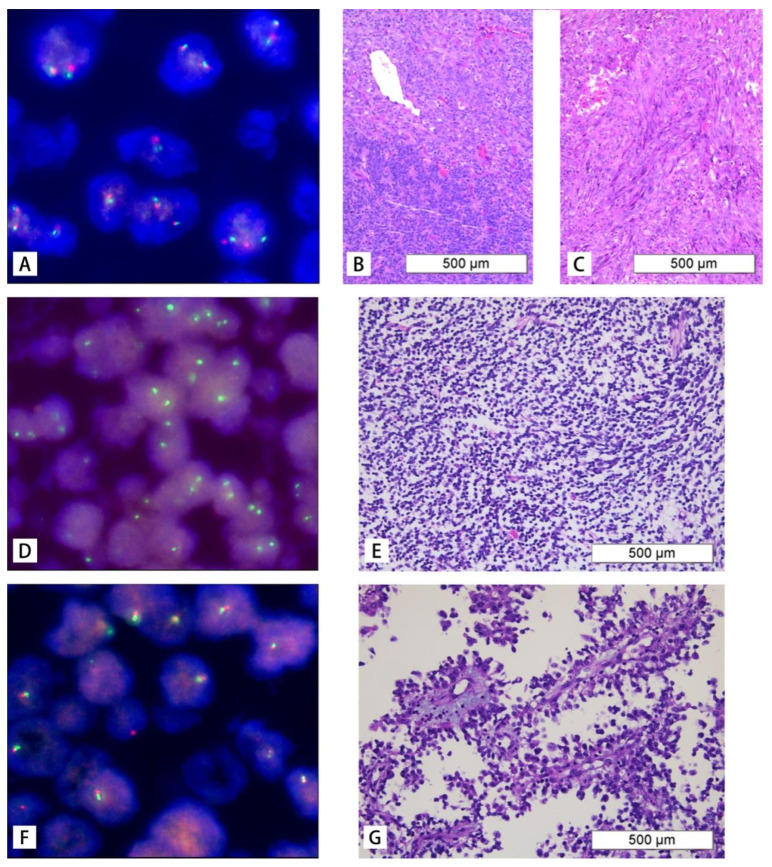
Fluorescence in situ hybridization (FISH) analysis of SWI/SNF-related matrix-associated actin-dependent regulator of chromatin subfamily B member 1 (SMARCB1) and corresponding hematoxylin and eosin (HE) staining. (**A**–**C**) No deletion. (**D**,**E**) Homozygous deletion. (**F**,**G**) Heterozygous deletion.

A SMARCB1 (22q11) gene deletion probe was used to label fifteen paraffin samples. Among these samples, 8 cases showed no deletion, while 7 cases had deletion, which included 1 case of homozygous deletion, 5 cases of heterozygous deletion, and 1 case of a special type of deletion. The non-deletion pattern was characterized by two red and two green signals (Figure 4A), and HE staining showed small round tumor cells arranged in chrysanthemum-like clusters (Figure 4B) or spindle-shaped bundles (Figure 4C). The homozygous deletion was characterized by a signal type of zero red and two green (Figure 4D), and HE staining presented as Figure 4E, whereas the heterozygous deletion was identified by a missing signal type of one red and one green (Figure 4F), with HE staining presented as Figure 4G. The special missing signal type was observed as two weak red and one green.

### 3.6. Following-Up

Twelve patients were followed up for a median of 36 months (range, 12–72 months); two children were alive, and the remaining ten had died. Most of the deceased patients received surgery alone without adjuvant treatment, whereas the surviving patients were still receiving postoperative chemotherapy at the last follow-up. The remaining six patients were lost to follow-up.

### 3.7. HE–IHC Dual-Path Model Performance

The performance of all models on the test set is shown in Table 2. The HE–IHC Dual-Path Model with transfer learning achieved the highest accuracy of 90.91%, significantly outperforming other control models. The HE–IHC Dual-Path Model without transfer learning reached an accuracy of 81.82%, which was 9.09% lower than the model with transfer learning. The single-path model using only immunohistochemistry images achieved an accuracy of 86.36%, while the single-path model using only histological images had an accuracy of 50.00%.

Further analysis of the misclassified cases showed that 3 out of 5 misclassified cases were atypical AT/RT without classical rhabdoid cells. The HE–IHC Dual-Path Model still correctly classified 2 of these 3 cases, demonstrating its value in assisting the diagnosis of atypical AT/RT.

### 3.8. Additional Independent Validation

To respond to the concern regarding the limited number of underlying AT/RT patients, we further evaluated the trained model on an additional independent cohort. This additional independent validation set included 175 newly collected histological and immunohistochemical images from six patients who were not involved in model development. The trained model correctly classified all 175 images, corresponding to an accuracy of 100% on this additional independent validation set. Although this additional validation cohort remained limited in size, it provided further support that the model was able to recognize shared diagnostic features in previously unseen patient material.

## 4. Discussion

The reviewer appropriately raised concerns regarding the limited number of underlying AT/RT patients and the existence of methylation-defined subgroups. In this context, the use of transfer learning and data augmentation was intended to reduce overfitting and improve robustness in a small-sample setting rather than to replace the need for validation [12,13,15]. Although AT/RT comprises molecularly distinct subgroups, these tumors still share diagnostically relevant image-level features discussed in this study, including rhabdoid morphology in a substantial proportion of cases, polyphenotypic immunohistochemical expression, and especially loss of nuclear INI1/SMARCB1 expression [16,17]. These shared patterns may explain why the model remained effective despite the limited cohort size. Importantly, in the additional independent test using 175 images from six newly collected patients, the model correctly classified all images. This supplementary result does not eliminate the limitation of sample size, but it does provide additional evidence that the model captured reproducible features beyond the original training cohort. Nevertheless, future studies should include larger multicenter cohorts with molecular subgroup annotation and balanced external controls to determine more rigorously whether performance is maintained across subgroup-specific variation.

AT/RT remains a diagnostically challenging embryonal tumor because of its morphological heterogeneity, polyphenotypic immunoprofile, and complex molecular alterations. In our cohort, most patients were very young, with 72.2% diagnosed before 3 years of age and 55.6% before 2 years of age, which is consistent with the recognized age distribution and aggressive clinical behavior of this disease [1,2,3]. Recent studies have further shown that histopathological patterns are related to molecular subgroup, and that immunohistochemistry continues to play an important role in practical molecular classification and laboratory diagnosis [4,5,6,16]. Case-based and practical pathology reviews have also emphasized that integrated clinicopathological assessment is essential for AT/RT diagnosis [18,19,20]. Therefore, diagnosis in daily practice still depends on careful integration of morphology, immunohistochemistry, and molecular testing rather than reliance on any single feature.

This point is especially important in pediatric pathology, where AT/RT may overlap morphologically with other embryonal tumors or present with only limited rhabdoid features. Classical rhabdoid cells with eccentric nuclei, prominent nucleoli, and abundant eosinophilic cytoplasm are diagnostically helpful when present, but a substantial proportion of tumors also contain primitive neuroectodermal, epithelial, or mesenchymal components. In our series, 12 cases showed classical rhabdoid morphology, whereas the remaining cases lacked typical rhabdoid cells and instead showed primitive neuroectodermal components with or without epithelial or mesenchymal differentiation. When the classical pattern is absent, awareness of this spectrum can help reduce diagnostic error and prompt timely ancillary testing.

Immunophenotypic diversity represents another important diagnostic feature of AT/RT. Consistent with previous reports, our cases commonly expressed epithelial markers such as CK and EMA, while neuronal or neural-related markers such as SYN and S-100 were observed in part of the cohort, and stromal markers including SMA or Desmin were only occasionally positive. GFAP or CD34 expression was also seen in a minority of cases. This polyphenotypic profile, together with loss of INI1 expression and a high Ki-67 proliferation index, supports the view that no single positive lineage marker is sufficient for diagnosis, whereas the overall immunophenotypic pattern is highly informative in routine practice.

Current evidence indicates that loss of INI1/SMARCB1 is central to AT/RT biology, but the underlying genomic events are heterogeneous. In routine diagnostic work, FISH remains a practical and commonly used molecular method for evaluating SMARCB1 deletion, and in this study it demonstrated both positive and negative probe patterns in tumors with loss of INI1 expression. Among the seven deletion-positive cases, only one showed homozygous deletion, five showed heterozygous deletion, and one showed a special deletion pattern, whereas eight cases showed no deletion signal by FISH despite loss of INI1 expression by immunohistochemistry. This finding further supports the complexity of molecular events in AT/RT. Accordingly, FISH results should be interpreted together with immunophenotypic and clinicopathological findings, particularly in cases with atypical morphology or discordance between protein expression and probe-based findings. In cases showing a negative FISH pattern despite INI1 loss, other molecular techniques may be required for further analysis [4,5,21].

The differential diagnosis also remains broad and includes other embryonal neoplasms as well as SMARCB1-deficient tumors. In this setting, retrospective analysis of a relatively large number of cases from a single center is valuable for improving understanding of the clinicopathological characteristics of AT/RT and may help reduce misdiagnosis in daily practice. Clinicopathological correlation therefore remains essential, because accurate distinction among these entities has direct implications for further molecular workup and clinical management [4,5,22].

AT/RT is a rare embryonal tumor of the pediatric central nervous system with histologically and immunophenotypically multiple phenotypic features, and it can be diagnosed by deletion of the SMARCB1/INI1 protein or, in rare cases, the SMARCA4/BRG1 protein in tumor cells. In addition, classification based on molecular features may provide a basis for future targeted therapy research. Recent studies have continued to support the clinical relevance of molecular grouping for risk stratification and outcome assessment [6,7,23], extending earlier molecular subgrouping work and consensus classification efforts [24,25].

The HE–IHC dual-path artificial intelligence model explored in this study supports the idea that combining histological and immunophenotypic information may improve pathological assessment. The developed model therefore provides a promising auxiliary diagnostic approach for AT/RT by integrating histological and immunological information and may be especially helpful in diagnostically challenging or atypical cases. However, its value should still be viewed cautiously, and broader external validation with larger multicenter datasets will be necessary before routine clinical implementation.

From a therapeutic perspective, AT/RT continues to pose major challenges because of its aggressive biological behavior and the very young age of many affected patients. These factors limit the tolerability of intensive treatment, especially radiotherapy, and underscore the need for more effective and less toxic targeted strategies in future studies. Previous studies have reviewed emerging targets, biology-based therapeutic approaches, and SWI/SNF-related mechanisms in rhabdoid tumors [26,27,28]. Recent work has identified subtype-related therapeutic vulnerabilities, drug-screening candidates, and additional molecular mechanisms that may support future treatment development [7,29,30,31,32].

## 5. Conclusions

AT/RT is a rare pediatric central nervous system embryonal tumor with diverse histological and immunophenotypic features, and SMARCB1 gene deletion shows complex patterns. The HE–IHC Dual-Path Model based on histological and immunohistochemistry images, combined with transfer learning and data augmentation, achieves high diagnostic accuracy (90.91%) and provides a reliable auxiliary tool for AT/RT diagnosis, especially for atypical cases. In addition, the model correctly classified all 175 images from an additional independent cohort of six newly collected patients, which provides preliminary support for its generalizability. This study not only enriches the understanding of AT/RT’s clinicopathological and molecular features but also bridges the gap between pathological research and clinical application through artificial intelligence technology.

## Figures and Tables

**Table 1:** Clinical and pathological characteristics of 18 pediatric atypical teratoid/rhabdoid tumor (AT/RT) patients, including SWI/SNF-related matrix-associated actin-dependent regulator of chromatin subfamily B member 1 (SMARCB1) status by fluorescence in situ hybridization (FISH).

Case	Age (Months)/Sex	Location	Treatment	Histopathology	Follow-Up	FISH (SMARCB1)
1	34/F	posterior fossa	surgery	small-blue-round cells	death	negative
2	15/F	temporal	surgery	rhabdoid cells, spindle cells	death	negative
3	46/M	spinal cord	surgery	rhabdoid cells	death	—
4	14/F	temporal	surgery	rhabdoid cells	death	negative
5	64/F	spinal cord	surgery	rhabdoid cells	death	special type deletion
6	38/M	posterior fossa	surgery	small-blue-round cells, epithelial components	loss of follow-up	—
7	10/M	cerebellum	surgery	small-blue-round cells	death	heterozygous deletion
8	38/F	cerebellum	surgery	small-blue-round cells, spindle cells	loss of follow-up	heterozygous deletion
9	79/M	cerebellum	surgery	rhabdoid cells	death	negative
10	28/M	basal ganglia	surgery	rhabdoid cells	death	—
11	6/F	spinal cord	surgery	rhabdoid cells, small-blue-round cells	death	negative
12	23/F	cerebellum	surgery, chemotherapy	rhabdoid cells, small-blue-round cells	death	heterozygous deletion
13	5/F	suprasellar	surgery	small-blue-round cells	loss follow-up	homozygous deletion
14	13/F	cerebellum	surgery	rhabdoid cells, small-blue-round cells	death	negative
15	16/M	frontotemporal	surgery, chemotherapy	rhabdoid cells, small-blue-round cells, spindle cells	death	negative
16	32/F	posterior fossa	surgery, chemotherapy	rhabdoid cells, small-blue-round cells	survival	heterozygous deletion
17	14/M	cerebellum	chemotherapy	rhabdoid cells, small-blue-round cells	survival	heterozygous deletion
18	14/M	posterior fossa	surgery	small-blue-round cells	loss follow-up	negative

**Table 2:** Test-set performance of the proposed hematoxylin and eosin–immunohistochemistry (HE–IHC) Dual-Path Model and control models.

Model	Accuracy (%)
Dual-path + transfer learning	90.91
Dual-path without transfer learning	81.82
Single-path immunohistochemistry images	86.36
Single-path histological images	50.00

## Data Availability

All slide images used in this study were completely de-identified to strictly comply with HIPAA privacy rules. Raw clinical and pathological data cannot be made publicly available because of ethical and privacy restrictions. Requests for access to de-identified image data, model code, and related experimental materials may be submitted to the corresponding author and will be considered upon reasonable request.

## Abbreviations

The following abbreviations are used in this manuscript:

AT/RT	Atypical teratoid/rhabdoid tumor
FISH	Fluorescence in situ hybridization
HE	Hematoxylin and eosin
IHC	Immunohistochemistry
HE–IHC	Hematoxylin and eosin–immunohistochemistry
GFAP	Glial fibrillary acidic protein
EMA	Epithelial membrane antigen
SMA	Smooth muscle actin
CK	Cytokeratin
INI1	Integrase interactor 1
SMARCB1	SWI/SNF-related matrix-associated actin-dependent regulator of chromatin subfamily B member 1
SP	Streptavidin–peroxidase
T1WI	T1-weighted imaging
T2WI	T2-weighted imaging
PyTorch	Python-based deep learning framework
